# Study on the correlation between carotid plaque calcification types and acute ischemic stroke

**DOI:** 10.3389/fneur.2025.1550014

**Published:** 2025-02-12

**Authors:** Tianyu Chu, Zhongping Guo, Yonggang Zhang, Ying Liu, Yan Gu

**Affiliations:** Department of Radiology, Lianyungang Clinical College of Nanjing Medical University, The First People's Hospital of Lianyungang, Lianyungang, China

**Keywords:** atherosclerosis, carotid plaque, calcified plaque, stroke, computed tomography angiography

## Abstract

**Introduction:**

Computed tomography angiography (CTA) was used to explore the correlation between the calcification types of carotid plaques and ipsilateral acute ischemic stroke. This could provide new insights into the clinical evaluation and treatment of ischemic stroke.

**Methods:**

This study obtained information on patients undergoing head and neck CTA examinations at the First People's Hospital of Lianyungang between September 2022 and August 2023 to investigate the clinical differences in baseline data between the acute ischemic stroke and control groups. Patients meeting the inclusion and exclusion criteria were classified into 1 to 6 groups according to their plaque calcification characteristics. The correlation between calcified plaque type and ipsilateral acute ischemic anterior circulation stroke was analyzed using paired sample chi-square and Spearman correlation tests.

**Results:**

Based on the inclusion and exclusion criteria, this study included 589 patients with plaques at the bifurcation of the carotid arteries. In both the acute ischemic stroke and control groups, sex, smoking, alcohol consumption, systolic blood pressure, blood sugar, cholesterol, high-density lipoprotein, and homocysteine levels were statistically significant (*p* < 0.05). During the Spearman correlation analysis between calcification type and acute stroke (1,178 carotid arteries), different calcification types of plaques were linked with ipsilateral acute anterior circulation stroke with statistically significant differences (*p* < 0.001). Finally, the chi-squared test of the paired samples showed that the grade of plaque calcification is often higher on the side of acute infarction than on the side without acute infarction, and the difference is statistically significant (*p* < 0.001).

**Discussion:**

Carotid plaque calcification is associated with acute ischemic stroke. Our findings provide novel insights into the study of calcification in carotid atherosclerotic plaques and additional radiological evidence to clinically assess the risk of ischemic stroke.

## 1 Introduction

Based on the latest statistical data, stroke is the second leading cause of death worldwide and the third leading cause of death and disability (1), possessing a heavy disease burden (2). Carotid atherosclerosis is a degenerative disease that can cause ischemia when plaques rupture (3), inducing severe neurological deficits. Calcification is universally present in atherosclerotic plaques; however, the underlying mechanism is not entirely understood. They often appear during atherosclerotic disease progression, where apoptotic cells, the extracellular matrix, and necrotic core substances become sources of calcium microcrystals. They can progress and fuse to form more extensive calcified deposits (4). However, research on the calcification inside plaques remains controversial. Calcified plaques are structural markers of plaque stability and protective factors against ipsilateral ischemic stroke, and are negatively associated with cerebrovascular diseases. However, recent studies have indicated that different calcification characteristics affect the plaque stability (5). Most studies have focused on a single characteristic of carotid plaque calcification. The classification method used in this study encompasses the size, location, and shape of the plaque calcification. Hence, six types of calcifications have been proposed by Saba et al. (6): type 1, no calcification inside the plaque; type 2, intima or superficial calcification; type 3, deep or large calcification; type 4, adventitial calcification with internal soft plaque thickness < 2 mm; type 5, mixed type with intima and significant calcification; and type 6, positive rim sign. The prevalence of cerebrovascular events was highest in patients with type 6 calcification.

The current study selected calcifications, elements which are easily observable in CT, to explore the correlation between each type of carotid plaque calcification and ipsilateral acute ischemic anterior circulation stroke according to the six calcifications types. This could provide a better radiological basis for evaluating carotid plaque vulnerability and early patient treatment.

## 2 Materials and methods

### 2.1 Study type

This is a single-center retrospective study.

### 2.2. Study subjects

#### 2.2.1. Study population

This study retrospectively obtained data from patients who underwent head and neck CTA examinations at Lianyungang First People's Hospital between September 2022 and August 2023. This study was reviewed by the Lianyungang First People's Hospital Medical Ethics (KY-20220726002-01).

#### 2.2.2. Inclusion criteria

Patients who underwent head and neck CTA for atherosclerotic plaques at the carotid bifurcation were included in this study. At the same time, the patient underwent an MR examination within a week.

#### 2.2.3. Exclusion criteria

The exclusion criteria included patients having incomplete imaging or clinical data. Non-intracranial atherosclerotic diseases, including aneurysms, vasculitis, Moyamoya disease, intracranial arterial dissection, reversible cerebral vasoconstriction syndrome, and vertebrobasilar dolichoectasia. Patients with suspected cardiogenic thrombus depicted by cardiac Doppler ultrasound or cardiac CTA, validated coagulation dysfunction, heart failure or respiratory failure, and renal dysfunction (serum creatinine > 133 μmol/L) were excluded. Furthermore, patients with severe disturbance of consciousness Intracranial hemorrhage, a history of cranial surgery, carotid stent placement, or carotid endarterectomy were excluded. Patients with a history of brain infarction having a diameter >1.5 cm (no hyperintensity in DWI) observed on MR were also excluded. The brain infarction could be seen in the posterior circulation supply area.

### 2.3 Examination plan and image analysis technology

#### 2.3.1 Scanning technology

##### 2.3.1.1. Equipment and reagents

A SIEMENS SOMATOM Definition Flash dual-source CT scanner was utilized, and the scanning parameters were set as follows: current, 125 mA; voltage, 100 kV; collimation, 16 × 0.6 mm, and slice thickness 0.75 mm. A venous catheter, double-pole release injector, and an iodixanol contrast medium (320 mg I/ml, Jiangsu Hengrui Pharmaceutical Co., Ltd., China) were used. A 320 mgI/ml contrast agent (iodoxanol, a non-ionic contrast agent with iodine) was administered using an 18-G catheter inserted within the antecubital vein at 3–5 ml/s, and 30 ml of physiological saline was injected at the same rate after injecting the contrast agent. The total amount and flow rate of the contrast agent were individualized according to the patient's weight and scanning range.

##### 2.3.1.2 CTA scanning protocol

None of the patients who underwent carotid CTA had a history of heart failure or contraindications for iodinated contrast agents. The patients were placed in the supine position, and CT coverage ranged between the aortic arch and carotid siphon. Patients were scanned from their feet to their heads. The images were tested before and after contrast agent injection. Scanning was performed from the lower edge of the aortic arch to the top of the skull. The scanning time was 8–12 s. Contrast agent tracing was applied to determine the CT value within the aortic arch or carotid level of interest. When the CT value was more than 100 HU, the scan was automatically triggered after a 4 s delay. After reaching the threshold, the patients were instructed to hold their breath and start scanning after a 4 s interval. Experienced radiologists conducted all imaging examinations. The patients were instructed not to swallow or move their head or body while scanning to obtain the desired vascular images using three-dimensional reconstruction technology.

#### 2.3.2 Image analysis technology

##### 2.3.2.1 Image analysis

Image analysis was performed on the improved phase of CTA examination inside the GPACS system (WW = 800, WL = 240).

##### 2.3.2.2 Carotid plaque calcification analysis

Two radiologists who had over 5 years of experience with head and neck imaging reviewed each image. Both reviewers were unaware of the patients' clinical data, and a third senior physician's opinion was sought in case of a disagreement.

The plaque at the carotid bifurcation was selected and evaluated as a whole. Calcification typing and plaque grouping were determined based on the morphology of the carotid plaque calcification by vision assessment.

##### 2.3.2.3 Carotid plaque calcification type

**Figure 1:**
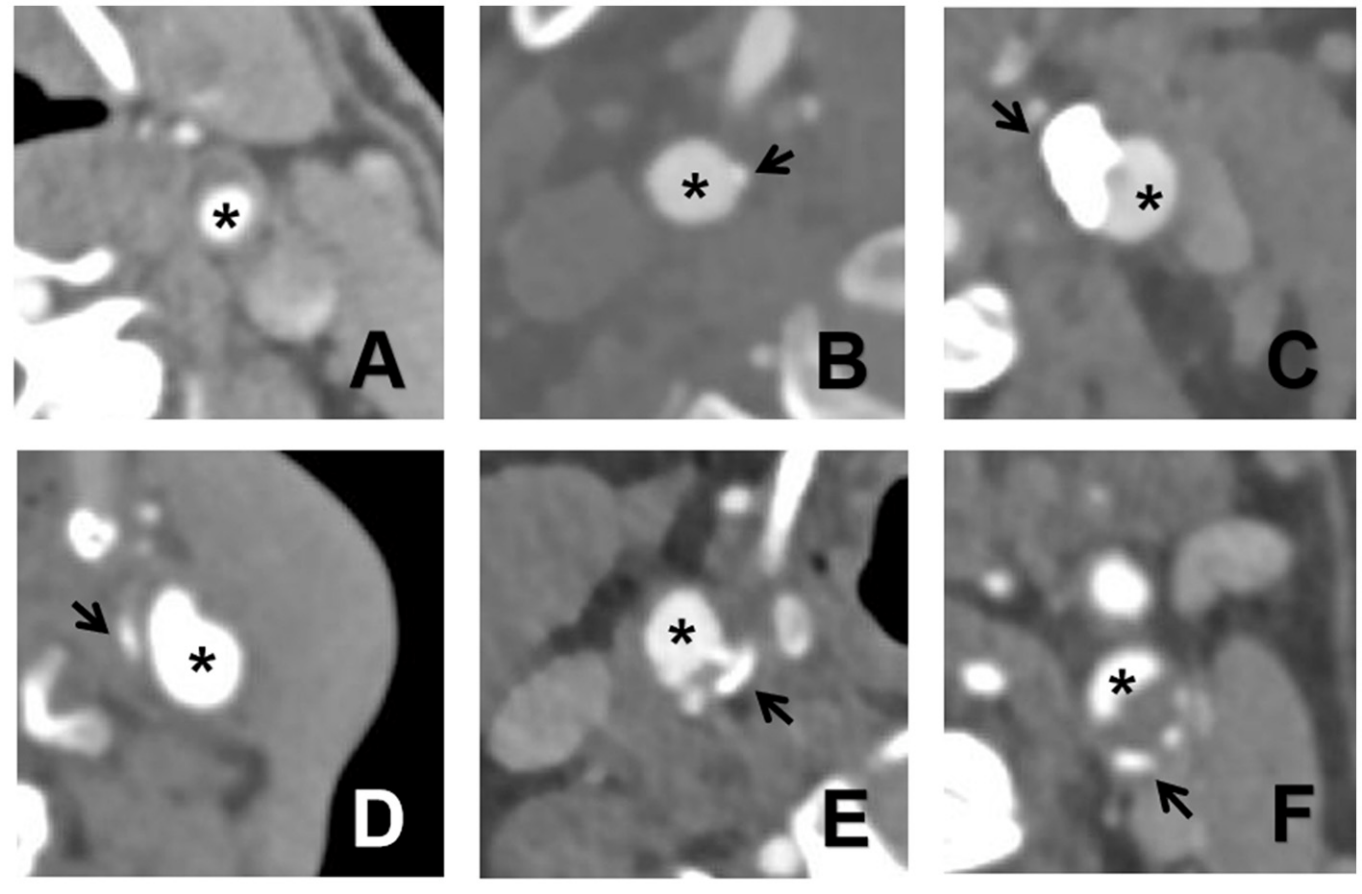
The six types of calcifications on CTA images. **(A)** no calcification inside the plaque; **(B)** intima or superficial calcification; **(C)** deep or large calcification; **(D)** adventitia calcification having internal soft plaque thickness < 2 mm; **(E)** mixed type with intima and significant calcification; and **(F)** positive rim sign. The asterisk indicates a lumen and the *arrow* indicates calcification.

The various calcification types on CTA images are depicted in Figure 1.

### 2.4. Demographic characteristics

Blood pressure, blood sugar, triglycerides (TG), total cholesterol (TC), low-density lipoprotein (LDL), high-density lipoprotein (HDL), lipoprotein A (LA), and homocysteine levels were measured while fasting in the morning for the first 48 h after admission. Medical history of diabetes, hypertension, coronary heart disease, and valvular heart disease. History of consumption of smoking and alcohol history.

### 2.5 Statistical methods

Data analysis was performed using SPSS version 26.0. Normally distributed continuous variables were expressed as the mean ± standard deviation, and analysis of variance helped conduct a comparison between groups. Quantitatively skewed distribution data are expressed as M (P25, P75). Moreover, a comparison between groups was performed using the Mann-Whitney U test. Categorical variables are expressed as frequencies and percentages. Additionally, the chi-square test was used to determine statistically significant differences between the groups. Kappa consistency testing was used to analyze inter-observer and intra-observer consistencies. *P-*values < 0.05 were considered statistically significant. Thus, Spearman's correlation analysis was used to analyze the correlation between the plaque type and acute ischemic anterior circulation stroke.

## 3 Results

### 3.1 Patient baseline data with carotid plaque calcification

After screening based on the predetermined inclusion and exclusion criteria, 589 patients, with plaques at the carotid bifurcation were included, with an average age of 65.3(30–90) years, including 351(59.6%) male patients. When analyzing the clinical baseline data between the acute and non-acute ischemic stroke groups, sex, smoking, alcohol consumption, systolic blood pressure, blood sugar, cholesterol, low-density lipoprotein, and homocysteine showed statistical significance (*p* < 0.05). The demographic and clinical data of the study population are presented in Table 1.

**Table 1:** Clinical baseline characteristics between the acute stroke group and the non-acute stroke group (*n* = 589).

	**The acute stroke group**	**The non-acute stroke group**	***P*-value**
Age (years)	67 (59, 74)	66 (58, 73)	0.233
Sex/male (*n*, %)	158, 65%	193, 55.78%	0.024
Smoking (*n*, %)	92, 37.86%	101, 29.19%	0.027
Drinking (*n*, %)	86, 35.39%	89, 25.72%	0.011
SBP, mmHg	148 (139, 161)	141 (130, 155)	< 0.001
DBP, mmHg	83 (78, 96)	81 (77, 93)	0.058
BS, mmol/L	5.34 (4.61, 7.66)	5.02 (4.45, 6.14)	0.001
TG, mmol/L	1.36 (1, 2.06)	1.31 (0.93, 1.80)	0.107
TC, mmol/L	4.70 (3.97, 5.53)	4.40 (3.50, 5.14)	< 0.001
LDL, mmol/L	3.02 (2.48, 3.57)	2.74 (2.06, 3.31)	< 0.001
HDL, mmol/L	1.12 (0.93, 1.32)	1.07 (0.91, 1.25)	0.134
LA, mg/L	177.7 (81.13, 341.10)	168.75 (87, 360.28)	0.799
Homocysteine, μmol/L	14.3 (11.3, 18.7)	12.8 (10.25, 16.70)	0.003

*P*-values < 0.05 were considered statistically significant.

SBP, Systolic blood pressure; DBP, Diastolic blood pressure; BS, blood sugar; TG, triglycerides; TC, total cholesterol; LDL, low-density lipoprotein; HDL, high-density lipoprotein; LA, lipoprotein A.

### 3.2 The correlation analysis between calcification types and acute stroke

**Figure 2:**
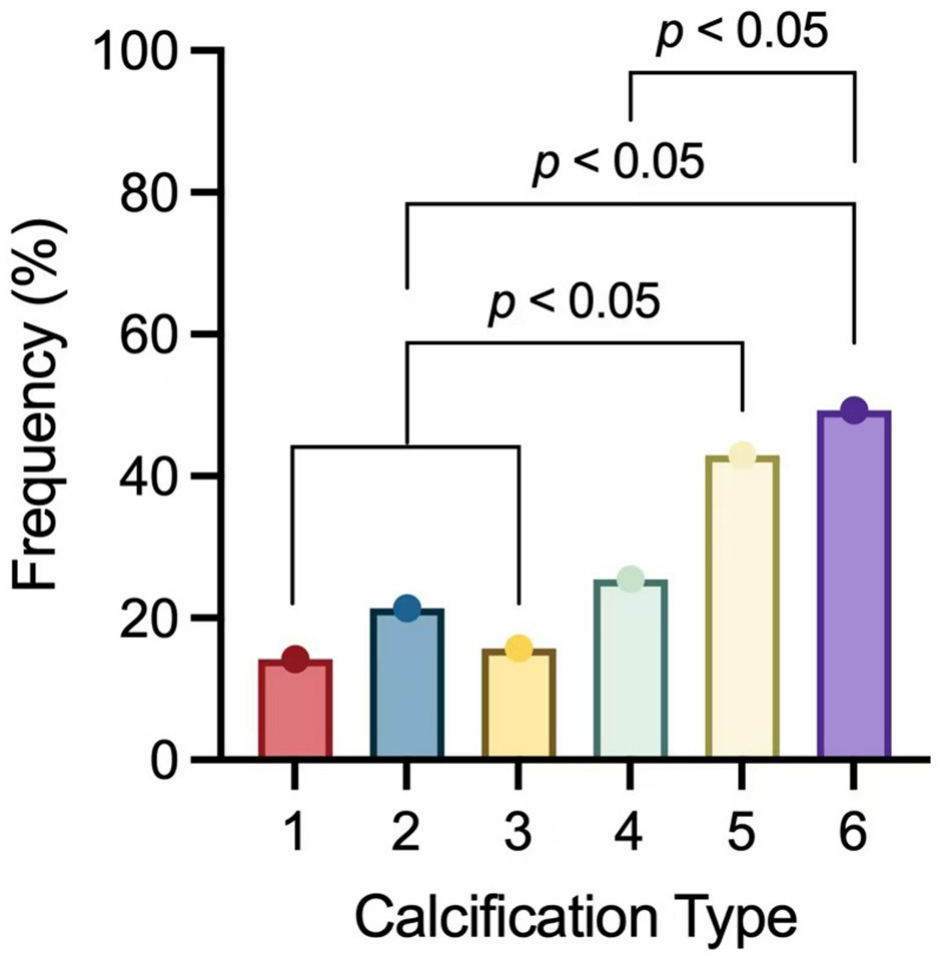
The incidence of ipsilateral acute stroke in type 1–6.

By observing 1,178 cases of carotid plaque and the occurrence of acute stroke in their blood supply area. We found that 68 cases in type 1 (68/478, 14.22%) had acute stroke, 54 cases in type 2 (54/253, 21.34%), 24 cases in type 3 (24/153, 15.69%), 26 cases in type 4 (26/102, 25.49%), and 52 cases in type 5 (52/121, 42.98%), 35 patients in the 6 type had acute stroke (35/71, 49.30%). Figure 2 presents the results of this study.

The Spearman test was used to analyze the correlation between the carotid plaque calcification type and ipsilateral acute anterior circulation infarction. Moreover, the results indicated a weak positive correlation between different calcified plaque types and ipsilateral acute anterior circulation stroke (*r* = 0.217, *p* < 0.001).

### 3.3 Paired sample chi-square test of carotid plaque calcification types and acute cerebral infarction

A paired-sample chi-square test was performed on the bilateral calcified plaque types in (227 patients) unilateral acute cerebral infarction. Calcified plaque types on the side with acute infarction were significantly higher than those on the side without acute infarction differences (*p* < 0.001). The results are presented in Table 2.

**Table 2:** Plaque calcification types on the acute and non-acute infarction sides of the same patient.

**The non-acute Infarction sides**	**The acute infarction sides**						
	**1**	**2**	**3**	**4**	**5**	**6**	**Total**
1	50	20	4	8	3	3	88
2	9	17	9	4	7	8	54
3	3	2	4	2	11	8	30
4	4	4	3	5	3	4	23
5	0	1	1	3	12	8	25
6	1	0	1	0	3	2	7
Total	67	44	22	22	39	33	227

## 4 Discussion

This study did not start with the traditional high-risk and low-risk plaques but focused on the more common calcification during clinical work. We grouped common calcified carotid plaques in clinical practice using CTA technology and developed a new classification method for calcified carotid plaques. We explored their associations with the occurrence of acute ipsilateral anterior circulation stroke. Type 6 calcified plaques are associated with acute ipsilateral anterior circulation strokes. This could help patients receive active and effective treatments before acute stroke.

Two radiologists who had been diagnosing head and neck images for more than 5 years reviewed the images in our study. Type 2 calcifications are easily distinguishable. In contrast, types 3 and 5 calcifications are difficult to distinguish. According to Kappa consistency testing, there was good inter-observer consistency (κ = 0.92).

First, the morphology of calcified plaques at the carotid bifurcation was examined, and the correlation between plaque calcification type and ipsilateral anterior circulation acute stroke was analyzed (considering 1,178 carotid arteries). Type 6 calcified plaques, that is, edge-sign-positive plaques, had the highest risk of ipsilateral anterior circulation acute stroke. Thirty-five of the 71 patients experienced acute ipsilateral anterior circulation stroke. This was confirmed by Spearman correlation analysis, which indicated a statistically significant correlation between the calcified plaque type and acute stroke. In the paired sample chi-square analysis, the carotid plaque calcification types on the side with acute stroke were often higher than those on the side without acute stroke. It was not difficult to identify the acute stroke proportion in type 6 calcified plaques, which was the most common type, by combining the overall proportion of each calcification type. The acute stroke prevalence in type 6 plaques in our study was much lower than the symptoms of type 6 calcified plaques observed by Saba et al. (6). This is because we introduced the definition of acute stroke. Based on the high signal intensity observed on DWI, false-positive patients were excluded. Simultaneously, patients with a history of larger infarctions were excluded, which reduced the incidence of false-negatives in the control group. In a retrospective cohort study, adventitial calcification with positive internal soft plaque edge signs was observed on CTA. This could predict intraplaque hemorrhage inside the carotid plaques (7). Kashiwazaki et al. observed that thin calcifications were associated with intraplaque hemorrhage, demonstrating a different clinical significance from that of thick calcifications (8), consistent with our research findings.

Gupta et al. observed that every increase of 1 mm can enhance the risk of stroke or transient ischemic attack by 2.7 times for soft plaques (9). Similar findings were made in our type 4 and type 6. Xu et al. reported intraplaque hemorrhage and lipid-rich necrotic cores combined with small punctate calcifications (10). Nandalur et al. discovered that the proportion of carotid plaque calcification, not the absolute volume, was associated with plaque stability. Specifically, the plaque may demonstrate better stability in some patients when calcification in carotid plaques accounts for more than 45% of the total plaque volume (11). However, similar results were not observed for type 1, 2, and 3. This is because calcification mechanisms and acute stroke are complex (12) and require further investigation. Type 5 refers to mixed type with intima and significant calcification and its complex mechanism needs to be further studied.

Secondly, our study identified that some baseline data between the acute and non-acute infarction groups showed significant statistical differences, which is consistent with previous studies. Sex, smoking, alcohol consumption, hypertension, hyperglycemia, and hyperlipidemia are the risk factors for ischemic stroke (13). Moreover, the consistency of systematic evaluations in observational studies (cohort and case-control studies) indicated a strong, positive, and dose-dependent correlation between serum total homocysteine concentration and stroke risk (14). Therefore, daily blood pressure, blood sugar, and blood lipid management while actively quitting smoking and alcohol consumption can help prevent the occurrence of stroke. However, patients depend on medications for disease management (15).

Similarly, we tried to exclude acute stroke caused by cardiac embolism or intracranial atherosclerotic plaques because baseline data inclusion and exclusion were performed by two experienced clinical doctors. However, patients with scattered lacunar infarctions identified by MR were not excluded. Because lacunar cerebral infarction has always been caused by cerebral small vessel disease (16), a common cause of cerebral small vessel disease is small artery sclerosis, which is a cerebral microvascular disease (17).

Our study had certain limitations. First, we could not avoid selection bias in the retrospective studies. Secondly, more complex plaques are difficult to describe using a single calcification type. Hence, typing carotid plaque calcifications is challenging.

This calcified plaque typing model must be validated using prospective and longitudinal cohort studies to establish its clinical reference value. The results of this study are clinically significant. A causal correlation between plaque calcification and acute stroke has not yet been established, although calcification is an accompanying phenomenon in atherosclerosis. However, the study results have established that the morphology of plaque calcification is associated with the occurrence of acute ischemic stroke.

## Data Availability

The raw data supporting the conclusions of this article will be made available by the authors, without undue reservation.
